# Scranton Type V Osteochondral Defects of Talus: Does one-stage Arthroscopic Debridement, Microfracture and Plasma Rich in Growth Factor cause the Healing of Cyst and Cessation of Progression to Osteoarthritis?

**DOI:** 10.5704/MOJ.2007.014

**Published:** 2020-07

**Authors:** N Singh, CR Pandey, B Tamang, R Singh

**Affiliations:** 1 Department of Orthopaedics and Traumatology, Grande International Hospital, Kathmandu, Nepal; 2 Department of Physiotherapy and Rehabilitation, Grande International Hospital, Kathmandu, Nepal

**Keywords:** scranton type V OCD, microfracture, talus, plasma rich growth factor, osteoarthritis

## Abstract

**Introduction:**

The study was conducted to evaluate the efficacy of arthroscopic debridement, microfracture and plasma rich in growth factor (PRGF) injection in the management of type V (Scranton) osteochondral lesions of talus and its role in healing the subchondral cyst and cessation of progression of ankle osteoarthritis.

**Material and Methods:**

This is a prospective case series conducted on patients who were diagnosed with type V osteochondral lesions of talus. All the cases were treated with arthroscopic debridement, microfracture, and PRGF injections. The patients were evaluated for the healing of subchondral cysts and progression of osteoarthritis with radiography (plain radiographs and computerised tomography Scan). Also, the patients’ outcome was evaluated with Quadruple Visual Analogue Scale, Ankle Range of Motion, Foot and Ankle Disability Index, Foot and Ankle Outcome Instrument and a Satisfaction Questionnaire.

**Results:**

Five male patients underwent arthroscopic debridement, microfracture and PRGF injection for type V osteochondral lesion of talus. The mean age of patients was 27.4 years (19-47 years). All the patients gave history of minor twisting injury. Subchondral cyst healing was achieved in all patients by six months post-surgery. However, four out of five patients had developed early osteoarthritic changes of the ankle by their last follow-up [mean follow-up 29 months (ranged 15-36 months)]. Despite arthritic changes, all the patients reported ‘Good’ to ‘Excellent’ results on satisfaction questionnaire and Foot and Ankle Disability Index and could perform their day to day activities including sports.

**Conclusion:**

Arthroscopic debridement, microfracture, and PRGF causes healing of the subchondral cyst but does not cause cessation of progression to osteoarthritis of ankle in type V osteochondral defects of talus. However, despite progress to osteoarthritis, patient satisfaction post-procedure is good to excellent at short-term follow-up.

## Introduction

Osteochondral defects of the talus is aseptic bone necrosis. The incidence of osteochondral defects (OCD) of the talus is 0.09 % in the literature with a prevalence of 0.002 per 100,000^1-3^. These lesions are of high clinical relevance as they are commonly missed in early phases when the radiographs appear normal. So, strong clinical suspicion is needed to diagnose these conditions.

Similar to the OCD of knee, elbow or any other joints, OCD of ankle also presents with pain in the early stages, more so with exertional activities. The management of ankle OCD ranges from conservative to surgical intervention^3^. The surgical interventions range from arthroscopic microfracture, drilling (with or without growth factor injections) to open treatment with bone grafts and augmentation procedures^3, 4^.

Various surgical treatment methods report favorable outcomes for treatment of OCDs of ankle. Arthroscopic debridement and bone marrow stimulation with or without platelet derived growth factor injections is one of the most common surgical treatment for OCD talus^5-7^. Based on the results of various studies, we conducted a prospective study evaluating the outcome of arthroscopic microfracture and Plasma Rich in Growth Factor injection [PRGF®-Endoret® - Parque Tecnológico de Álava, Leonardo Da Vinci, 14B 01510 Miñano (Álava), Spain] in the treatment of OCD type V (described by Scranton and McDermott)^8^.

## Materials and Methods

**Fig. 1:**
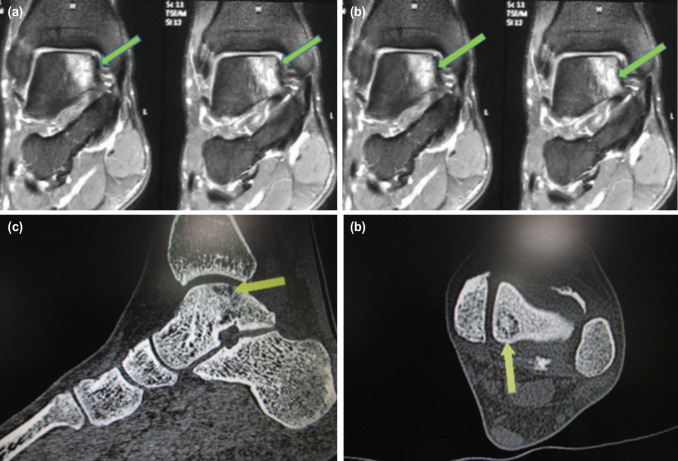
Imaging for diagnosis of OCD type V (a, b) magnetic resonance imaging; T1 and short-tau inversion-recovery (STIR) sequence respectively depicting the site of OCD; (c, d) CT scan images showing subchondral location of cyst). Green and yellows arrows depict the lesion.

Ours is a prospective case series conducted during the period of July 2014 to July 2017 at a tertiary care center. All the cases above 16 years of age, who were diagnosed type V OCD clinico-radiologically, and were treated with arthroscopic debridement, microfracture and Plasma Rich in Growth factor (PRGF) injection were included in the study. Other inclusion criteria were no history of prior ankle surgeries or other associated injuries and a minimum follow-up of one year. Cases of OCD other than type V or those treated with other methods (in contrast to the above mentioned method) or those without a follow-up of at least one year were excluded from the study. Pre-operatively, all the cases were evaluated and confirmed for OCD type V by history, clinical examination, radiographs, computerised tomography (CT) ccan and magnetic resonance imaging (MRI) (Fig. 1). After we confirmed the diagnosis, the patients were counseled regarding the surgical procedure and its prognosis and a consent was taken.

**Fig. 2:**
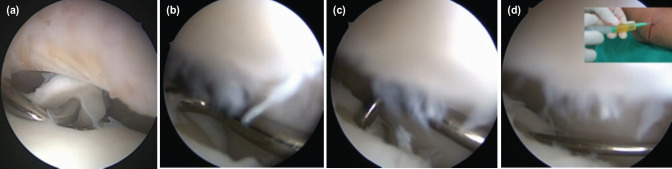
Shows arthroscopic procedure; (a) loose cartilage flap, (b) subchondral area of bone after debridement, (c) microfracture, (d) PRGF-Endoret® injection into the holes of microfracture with needle inserted in one of the holes under arthroscopy guidance.

After obtaining clearance for surgery, all the patients underwent surgery and were operated by a single surgeon and his team. All the surgeries were done under spinal anaesthesia and tourniquet was used in all the cases. All patients received single dose of intravenous cefazolin 1 gram half an hour before surgery. Supine position with heel placed at the end of the table was used in all the cases. This allowed free plantar and dorsiflexion of the ankle. The distraction of joint was achieved by direct manipulation (traction) by first assistant. The portals used were anteromedial and anterolateral. In all cases, anteromedial portal was made first and then, the anterolateral portal was made under arthroscopy guidance. A 4-mm, 30-degree angled arthroscope was used in all cases. In cases with difficult visualisation due to synovial hypertrophy, partial synovectomy was done to clear the joint. This was followed by an arthroscopic tour of the whole ankle joint. The area of OCD which was localised pre-operatively, was then probed to identify any cartilaginous loosening or tear. In case of cartilage loosening or tear, the flap was removed until normal cartilage was found at the margins. Debridement was followed by micro fracture and PRGF injection through the holes of microfracture. In cases, where microfracture holes were inaccessible with needle, it was directly injected into the joint. The surgical procedure has been depicted briefly in Fig. 2. Tourniquet was released only after closure of portals and application of compression bandage. No post-operative splint was given. The dressing was done on the first post-operative day, and patient was discharged.

Immediate follow-up was done on 4th, 9th and 14th post-operative days with suture removal on 14th post-operative day. After that, follow-up was done at six weeks, three months, six months, one year and successively every year. CT scan was done at six weeks, three months, six months and one year to monitor the healing of the subchondral cyst. Two additional doses of PRGF injections were injected at one week and two weeks post-operatively from the day of surgery. Ankle range of motion was initiated in immediate post-operative period along with static strengthening exercises. Weight-bearing walking was allowed only after six weeks. Patients were cleared for sporting activities after six months post-surgery and complete healing of the cyst. Outcome measures used were quadruple Visual Analogue Scale (VAS), Satisfaction Questionnaire (SQ) for ankle function on a scale of 1–6 (1-very good; 2-good; 3-satisfied; 4-sufficient; 5-insufficient; 6-poor)^9^, Foot and Ankle Disability Index (FADI)^10^, Foot and Ankle Outcome Instrument (FAAOS)^11^, ankle Range of Motion (ROM) measured by goniometer and radiographs yearly. Of the above mentioned measures, VAS, FADI and ankle ROM were also measured pre-operatively in order to determine whether there was significant improvement in outcome.

## Results:

**Fig. 3:**
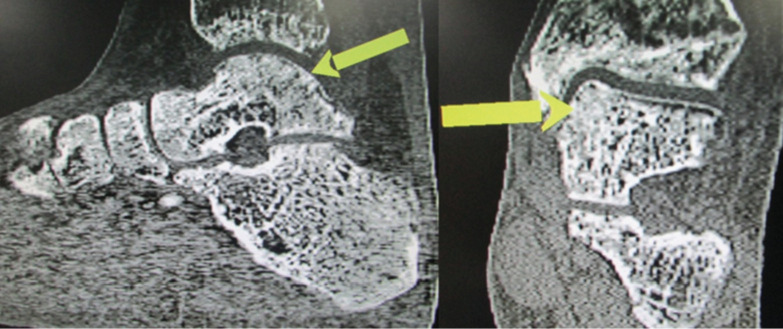
CT scan images showing healing of the subchondral cyst (Arrows- healed areas of subchondral cyst).

During the period of study, we surgically treated six cases (all males) of OCD type V with arthroscopic ankle joint debridement, microfracture and PRGF injection intra-operative and then two additional doses at weekly intervals. We only included five cases since one of the cases did not meet the inclusion criteria. The mean age of patients was 27.4 years (ranged 19-47 years). The average duration of symptoms before surgery was 10.6 months. All the cases gave history of minor twisting injury of ankle before the symptoms started for which they did not seek any consultation. All the lesions were located on the posteromedial part of the talus. The demographic details of these patients have been in Table I. None of the patients had any post-operative wound complication or additional comorbidities. All the patients started walking by six weeks (partial weight bearing) with crutches which was gradually progressed to full weight bearing. All the patients returned to their normal day to day activities by three months, and sportsperson to their respective sports by six months. Serial CT scan at six weeks, three months and six months showed complete healing of the cystic lesion by six months post-operatively (Fig. 3).

**Table I:** Showing Demographic details of the patients. (+: Present; Y: Yes; N: No; M: Male)

Case No.	Age (in years)	Sex	History of twisting	Profession	Duration of Symptoms (Months)	Follow-up (in months)	OA Changes on Radiogeaphs
1	47	M	+	Businessman	9	36	Y
2	22	M	+	Footballer	10	30	Y
3	19	M	+	Student	12	28	N
4	23	M	+	Army Professional	9	36	Y
5	26	M	+	Student	13	15	Y

**Fig. 4:**
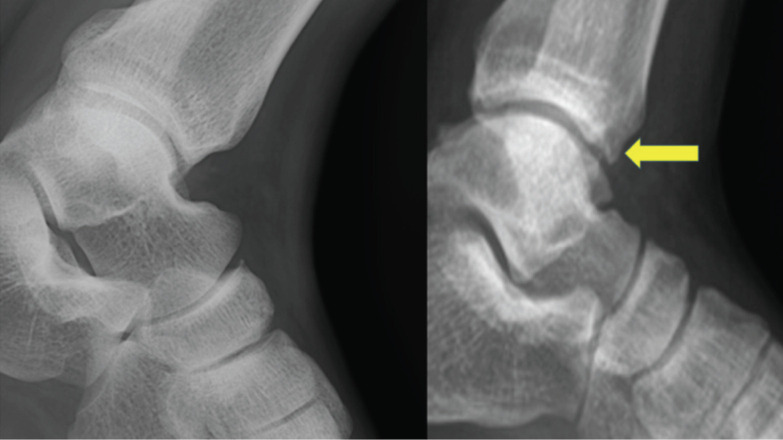
Showing degenerative changes in ankle joint at latest follow-up. Arrow showing anterior and posterior lipping along with sclerosis of subchondral bone (grade 1 OA changes).

**Fig. 5:**
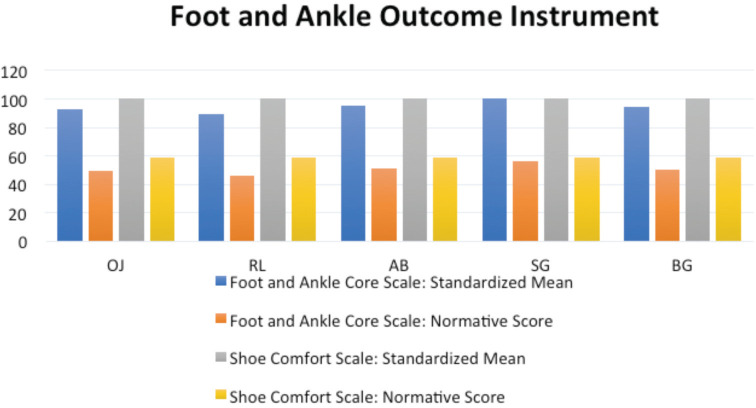
Results of Foot and ankle Outcome Instrument (American Academy of Orthopaedic Surgeons) of the patients on latest follow-up.

**Fig. 6:**
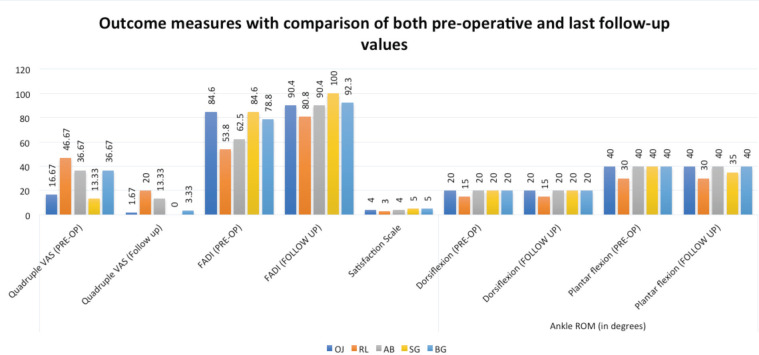
Outcome of the patients based on Foot and Ankle Disability Index (FADI), Ankle Range of Motion (ROM) and Quadruple Visual Analogue Scale (VAS).

However, four out of five patients developed early osteoarthritic changes (without collapse of bone) at their last follow-up which was detected radiologically by doubtful joint space narrowing and possible osteophytic lipping of the joint (Kellgren and Lawrence Grade 1) along with sclerosis of subchondral bone^12^ (Fig. 4). There was no limitation of day to day activities. The fifth patient did not have any osteoarthritis (OA) or bony collapse at 15 months follow-up. The results of outcome scores have been shown in Fig. 5 and 6. Mean follow-up of patients was 29 months (ranged 15-36 months). Upon comparison between pre-operative and latest follow up values of quadruple VAS, FADI and ankle ROM, there was significant improvement in VAS at follow-up (p-value=0.003848) and FADI at follow-up (p-value=0.0131). With regards to ankle ROM, there was no significant difference for dorsiflexion (both pre-operative and follow-up values were same for all the subjects) and plantarflexion (p-value=0.3739). Satisfaction questionnaire revealed a median value of 2 signifying that the majority of the patients felt that the outcome of the procedure was good.

## Discussion

Only a handful of papers have discussed elaborately about etiology, classification, diagnosis, management, and outcome of OCD talus^3, 13-15^. The relative rarity of the occurrence of these lesions may be explained by the fact that these lesions are often undiagnosed by treating physicians due to the lack of acquaintance with the condition and also because of late presentation of the patients. The reason for late presentation to doctors may be due to lack of history of significant trauma or in few cases only history of minor trauma (twisting)^3, 13, 16^. Up to 6.5% of the cases occur following ankle distortion^3, 17, 18^. The mechanism of pronation or supination with or without ligament ruptures has been commonly associated with it^3, 16, 19-21^. Many of these patients tend to neglect the minor twisting injuries and do not visit a doctor for treatment and by the time they present, these lesions would have already progressed to osteoarthritis (OA) of ankle. In all our cases, we could find a preceding history of minor twisting injury and all occurring in physically active individuals. So, we suggest that any case of persistent ankle pain following minor twisting injury should prompt for the evaluation for OCD. The strong suspicion is needed to diagnose these lesions.

Several classification systems have been proposed to describe different stages of OCD of talus^8, 13, 20, 22^. Berndt and Harty^20^ were the first to describe the various types of OCD based on radiograph and it is still the most popular classification in use^13^. Loomer *et al* added an additional group, type V (based on radiology, predominantly CT scan) in which there is an underlying cystic lesion in subchondral bone^22^. These type V OCDs had intact overlying cartilage according to their classification. In our study, however, we found out on radiological evaluation that these subtypes may or not have cartilage abnormality (loosening to frank tear and displacement). Thus, some of these types with an inherent loosening of cartilage cannot be labelled as type V according to classification given by Loomer *et al*^22^. Another classification proposed by Ferkel *et al*^23^ based on CT scan has described the lesions similar to our cases under stages 1 and 2A. The classification by Scranton and McDermott is more comprehensive and describes these subchondral cysts in type V of their classification which is more appropriate to describe lesions seen in our case^8^. Thus, our case series is based on the management of type V as described by Scranton and McDermott. These computed tomography-based classification can be used in adjunct with Berndt and Harty classification to describe all the talar OCDs appropriately.

Both conservative and surgical management have been tried for managing OCDs with conservative management preferred for early stages according to Berndt and Harty classification^13^. Though conservative management is advised, researches have proven it to be associated with the poorer outcome as compared to the surgical management^24^. We also suggest that surgical management is the better of the options to treat these lesions. According to Zwingmann *et al*, 75% of the patients had “good” to “excellent” outcome with surgical management which was stage independent. Literature mentions 14 different treatment concepts for various types of OCD lesions^3^. The primary goal of surgical management is to either regenerate or to fill the defect and restore the articular surface to prevent osteoarthritis apart from reducing pre-operative symptoms^3^.

Among the surgical managements available, arthroscopic debridement and bone marrow stimulation yields approximately 85% success. In case of failure of the above mentioned procedure, other options include osteochondral autograft transfer, autogenous cancellous bone graft, and autologous chondrocyte implantation^25^. Following the more commonly accepted treatment, we too performed arthroscopic debridement, microfracture and PRGF injection through the microfracture holes into the cyst. All our cases in this study had type V lesions located on the posteromedial aspect of the talus with associated cartilage loosening or non-displaced tear. The subchondral cystic lesion was the predominant lesion, and hence the goal was to decompress the cyst and promote healing in order to prevent collapse and future degeneration of the joint. PRGF was used to enhance healing of the cyst and cartilage defect. Retrograde drilling was another option, if we had found intact cartilage on arthroscopic evaluation, in order to prevent damage to the overlying cartilage since a success rate of 81-100% has been reported with this method^25^. However, all our cases had either loosening or tear (displaced or non-displaced) of the cartilage and thus, we decided to debride the cartilage and perform microfractures instead of retrograde drilling. We preferred this technique for our cases because of its low complication rate, relative ease of performing the procedure and high success rate as reported in the literature^25^.

As mentioned earlier, the main principle of our treatment was to improve the functional outcome as compared to preoperative period and to halt or delay the progression to degenerative OA of ankle. However, four out of five of our patients developed early OA changes within three years of the treatment (80%) in contrast to 33-34% on long-term follow-up reported in literature^5^.

A recent study conducted by Seo *et al*^26^ mentions that none of their cases out of total 142 patients developed osteoarthritis at long-term follow-up with conservative management. Our study in contrast reports 80% OA rate when the surgical technique reported by us is chosen. The reason for this rapid progression, as explained by Reilingh *et al*^5^, is due to the formation of fibrocartilage which decreases in quality over time and shows inferior wear characteristics, and also due to irregularity and depression in subchondral bone plate after debridement of cartilage and microfracture. The rapid progression in our cases might be because of denuding normal hyaline cartilage during surgery Also, we believe that since all our cases were Scranton type V in contrast to the study by Seo *et al*^26^ where majority of their patients are types 1 and 2 and they have not considered Scranton type V in their study, led to marked difference in outcome in terms of OA.

Though there was a progression to early OA in most of the cases with this method, there was healing of the subchondral cyst in all the cases within six months post-operatively when followed up by CT scan. Also, the satisfaction level was good to excellent in all the patients despite the onset of OA. The nature and location of minor ankle discomfort which they complained of was different than that of pre-operative period; with discomfort being more generalised around ankle rather than being localised deeply around medial aspect. The overall outcome was ‘good’ to ‘excellent’ when judged by various outcome measures as mentioned in Fig. 5 and 6, and also when comparing the pre-operative and latest follow-up VAS and FADI, there was statistically significant improvement in both with no deterioration of ankle ROM.

We have considered radiological signs for early osteoarthritis (Grade 1) in our evaluation because, based on previous study conducted by Hart and Spector^27^, 62% of these so called ‘doubtful lesions’ would progress to advanced OA over 10 years, and hence patient should be counselled accordingly despite relief in symptoms. We believe the symptoms were relieved because of formation of fibrocartilage as mentioned by Reilingh *et al*^5^.

The major limitation of our study is the number of cases in the study. However, given the rarity of these lesions, we could still draw out some conclusions. Further larger studies including randomised controlled trials are needed to confirm the findings from our study.

## Conclusion

We conclude that type V OCDs may have associated cartilage damage which will present itself in due course of time. Arthroscopic debridement, microfracture and PRGF injections do not halt or delay the progression of OA ankle in type V OCD but helps in the healing of subchondral cyst. This surgery can be undertaken to alleviate the pre-operative symptoms of OCD, but counselling is needed for future probability of OA. Also, we conclude that no single classification system can define all the OCDs at present, and comprehensive classification merging existing classification systems with the proposed management of the stages is needed.
